# Fine Particulate Matter (PM_2_._5_) Promotes CD146 Expression in Alveolar Epithelial Cells and *Cryptococcus neoformans* Pulmonary Infection

**DOI:** 10.3389/fmicb.2020.525976

**Published:** 2021-01-18

**Authors:** Zhixiao Sun, Ningfei Ji, Jingxian Jiang, Yuan Tao, Enrui Zhang, Xiaofan Yang, Zhengxia Wang, Zhongqi Chen, Mao Huang, Mingshun Zhang

**Affiliations:** ^1^Department of Respiratory and Critical Care Medicine, The First Affiliated Hospital of Nanjing Medical University, Nanjing, China; ^2^NHC Key Laboratory of Antibody Technique, Department of Immunology, Nanjing Medical University, Nanjing, China; ^3^Laboratory Center for Basic Medical Sciences, Nanjing Medical University, Nanjing, China

**Keywords:** PM_2_._5_, CD146, *Cryptococcus neoformans*, lung, aryl hydrocarbon receptor

## Abstract

Air pollution is a leading cause of increasing infectious lung diseases. Pulmonary cryptococcosis is a fatal fungal pneumonia in acquired immunodeficiency syndrome patients. In some cases, the pathogen *Cryptococcus neoformans* also develops dormant nodules in immunocompetent individuals. In the present study, we demonstrated that fine particulate matter (PM_2_._5_) increased CD146 expression in alveolar epithelial cells and promoted *C. neoformans* pulmonary infection. Aryl hydrocarbon receptor (AhR) signaling was required for increased expression of CD146 in epithelial cells treated with PM_2_._5_. In a murine model of pulmonary infection, PM_2_._5_ promoted fungal infection, and CD146 deficiency decreased the fugal burden of *C. neoformans*. Our study may highlight the importance of air pollution to lung mycosis and CD146 as a target for preventing infectious lung diseases.

## Introduction

Exposure to air pollution is a great threat to human health. New data from the WHO estimate that 9 out of 10 people breathe polluted air. In particular, fine particulate matter with an aerodynamic diameter of less than 2.5 μm (PM_2_._5_) may cause 7 million deaths worldwide every year (Orru et al., 2017). Once inhaled, PM_2_._5_ deeply penetrates into pulmonary tissues, leading to various cardiopulmonary diseases, including lung cancers (Fu et al., 2015), chronic obstructive pulmonary diseases (COPD) (Wen and Gao, 2018), and respiratory infections. Bacteria rank as the most abundant microbes attached to PM_2_._5_ (Cao et al., 2014). As expected, bacterial pneumonia is associated with an increase in air pollution (Croft et al., 2019). PM_2_._5_ also exaggerates acute virus respiratory infections (Horne et al., 2018; Croft et al., 2019). Although fungi are ubiquitous in the environment (Andualem et al., 2019), polluted air-associated fungal pneumonia and the mechanisms are largely ignored.

As environmental fungi are ubiquitous, most people have been exposed to pulmonary fungal pathogens. Among the fungal pneumonia, pulmonary cryptococcosis is caused mainly by *Cryptococcus neoformans*. As a common environmental encapsulated fungus found in soil (Emmons, 1951) and bird feces (Soltani et al., 2013), *C. neoformans* infects approximately 70% of children >5 years old without evident clinical symptoms (Goldman et al., 2001). In addition, *C. neoformans* may cause fatal pulmonary infection in immunocompromised patients. Globally, over 180 thousand AIDS patients may die annually from *C. neoformans* infection, which was worse especially in sub-Saharan Africa without effective therapy (Rajasingham et al., 2017). Airborne *C. neoformans* from aerosolized fungi-contaminated soil is capable of deep lung deposition (Neilson et al., 1977). Alveolar epithelial cells lining most of the internal surface of the lungs are the first targets for the establishment of *C. neoformans* pulmonary infection (Taylor-Smith, 2017). We hypothesized that PM_2_._5_ influenced alveolar epithelial cells and aggravated *C. neoformans* pulmonary infection.

Originally identified as a tumor marker for melanoma (MCAM) (Wang and Yan, 2013), CD146 is actually involved with diverse diseases. For example, CD146 in macrophages mediates cell adhesion and foam cell formation in atherosclerosis (Luo et al., 2017). Th17 cells expressing CD146 contribute to inflammatory response in systemic sclerosis (Gabsi et al., 2019). CD146 is also associated with pulmonary infections, in which it promotes the adherence of bacteria or viruses to airway epithelial cells (Simon et al., 2011; Wu et al., 2013; Berman et al., 2014, 2016). The roles of CD146 in fungal infection still remain largely elusive. Adhesion of *C. neoformans* to epithelial cells is the first step to infection onset (Taylor-Smith, 2017). We hypothesized that PM_2_._5_ increased CD146 expression in alveolar epithelial cells, therefore increasing *C. neoformans* adhesion and pulmonary infection.

## Materials and Methods

### PM_2_._5_ Sampling and Analysis

PM_2_._5_ was collected by a Laoying 2030 air sampler (Laoshan Institute of Applied Technology, Qingdao, China) in Taizhou. The collection was adhered to Teflon-coated quartz fiber filters and then cut into pieces. The pieces were washed with PBS three times on ice and filtered again by a Falcon 40-μm strainer (Corning, NY, United States). The filtrate was freeze-dried in a vacuum and resuspended in PBS at a concentration of 5 mg/ml. PM_2_._5_ was finally stored at −20°C.

By analysis with a Waters Alliance e2695 HPLC system connected to a Waters 2489 UV/Vis Detector (MA, United States) in a laboratory at Taizhou Environmental Monitoring Center (Jiangsu, China), polycyclic aromatic hydrocarbon (PAH) complexes were determined to be the main component of PM_2_._5_. The analysis followed a standard protocol for the determination of particulate phase PAHs (HJ647-2013, Ministry of Environmental Protection, China) (Chen et al., 2019).

### Animals

Female C57BL/6J mice at 6–8 weeks of age were purchased from the laboratory animal center, Nanjing Medical University (Nanjing, China). CD146 knockout (KO) mice (female, 6–8 weeks old) with a C57BL/6J background were purchased from Cyagen, Suzhou, China. All mice were housed in a specific pathogen-free (SPF) environment and provided plenty of water and food. All experiments with animals were approved by the Nanjing Medical University Ethics Committee (1708004).

### Cell Culture

A mouse pulmonary epithelial cell line (MLE-12) was purchased from the ATCC (VA, United States) and cultured in DMEM (Gibco, United States) containing 10% fetal bovine serum (FBS, Gibco, United States), 100 IU/ml penicillin, and 100 μg/ml streptomycin (Hyclone, United States) in a 5% CO_2_ atmosphere at 37°C. MLE-12 cells were treated with PM_2_._5_ at specific concentrations or for specific durations after seeding in 24-well plates (Thermo, United States) or glass-bottom dishes (Thermo, United States).

Primary alveolar epithelial cells from mice were purified using 0.1% collagenase, 0.25% trypsin, and DNase I and selected with mouse IgG (36111ES60, Yeasen, China) as described in the literature (Nabhan et al., 2018). Briefly, PBS was injected into the right ventricle of the mice to flush the blood. Then 0.1% collagenase and 0.25% trypsin was injected into the trachea before the whole lung was isolated. After incubating in 0.1% collagenase and 0.25% trypsin for 20 min, the lung was placed in DMEM with DNase I to continue oscillating. Finally, the lung tissue was ground through the cell sieve and was selected by mouse IgG.

### Fungal Culture

*Cryptococcus neoformans* H99 (#208821) was purchased from ATCC. Acapsular strain CAP59 was kindly provided by Dr. Min Chen in the Second Military Medical University (Yang et al., 2019). The fungi were cultured in Sabouraud dextrose broth (Becton Dickinson, United States) at 32°C with gentle rotation for 16–18 h. After washing with PBS, the amount of fungi was quantified by a cell counting chamber.

### Adhesion Assay

MLE-12 cells or primary alveolar epithelial cells were incubated to form a monolayer in 24-well plates. The cell monolayer was treated with PM_2_._5_ (10 μg/ml) or PBS for 12 h. After washing with PBS, 1 ^∗^ 10^6^ *C. neoformans* were added to the culture medium for 4 h. After the supernatant was removed, the wells of cell culture plates were washed with 1 ml PBS, preheated at 37°C. PBS was slowly added into the wells along the wall of each well, and the 24-well plate was gently shaken for 30 s. After sucking up the liquid, the abovementioned process was repeated twice. Autoclaved distilled water was added for 20 min to lyse cells and re-suspend fungi. Appropriate dilutions of the re-suspension were plated onto Sabouraud dextrose agar plates and cultured at 32°C for 48 h to count CFUs.

*Cryptococcus neoformans* were stained with FITC (1 mg/ml, Sigma-Aldrich) for 30 min (Yang et al., 2019) and added into the culture medium of MLE-12 cells pretreated with PM_2_._5_ or PBS. After 4 h, the MLE-12 cells were extensively washed three times with PBS and visualized by an Olympus IX73 fluorescence microscope.

### Pulmonary Fungal Infection Model

The mice were intranasally administered with PBS or 10 mg/kg PM_2_._5_. After 24 h, the mice were intranasally infected with 1^∗^10^6^ *C. neoformans* in 40 μl of PBS. After 4 h, all mice were anesthetized to collect lung and bronchoalveolar lavage fluid (BALF) (Han and Ziegler, 2013). The bronchi of the mice were irrigated slowly and repeatedly three times with 0.5 ml PBS with 0.1 M ethylenediaminetetraacetic acid (EDTA) to collect BALF, and then lung tissues were collected after the hearts of the mice were perfused (Zhao et al., 2014; Heyen et al., 2016). The right inferior lobar bronchus was used for histological staining, and the left lobe was ground for CFU assay or ELISA analysis. The left lung lobes of the mice in 1 ml of PBS were ground at 60 HZ for 5 min by Tissuelyser-24 (Jingxin, China). The homogenates were diluted and plated on Sabouraud dextrose agar plates, and CFUs were counted after 48 h at 32°C. Then the homogenates were centrifuged to collect the supernatant for ELISA analysis.

### Cell Transfection

MLE-12 cells seeded in cell plates were incubated overnight before transfection. A CD146 expression plasmid, an siRNA plasmid, or the corresponding blank vehicles (Abmgood, China) were mixed with Lipofectamine 3000 (Invitrogen, United States) in DMEM without FBS, penicillin, and streptomycin for 25 min and were then transfected into MLE-12 cells at 60–80% density in DMEM for 48 h. PM_2_._5_ was used to treat MLE-12 cells for 12 h, and total protein was then extracted.

### Western Blotting

The cells or tissues were lysed with RIPA buffer (89900, Thermo, United States) containing protease and phosphatase inhibitors (78443, Thermo, United States) on ice for 20 min, and the lysate was then centrifuged for 10 min to collect the supernatants into a new Eppendorf (EP) tube. Then, a bicinchoninic acid (BCA) assay (P0012S, Beyotime, China) was used to measure the concentrations of proteins. The proteins were separated by 10% SDS-PAGE and transferred to polyvinylidene difluoride (PVDF) membranes at 300 mA for 135 min. The PVDF membranes were blocked with 5% skim milk powder for 1 h at room temperature, incubated with each primary antibody (Table 1) at 4°C overnight and then incubated with goat anti-rabbit IgG HRP-conjugated (EarthOx Life Sciences) or goat anti-mouse IgG HRP-conjugated (EarthOx Life Sciences) for 1 h at room temperature after washing four times with TBST for 5 min each time. After the PVDF membranes were washed four times with TBST for 7 min each time, the specific antibody-bound proteins were visualized with Immobilon Western Chemiluminescent HRP Substrate (Millipore, MA, United States) and a G:Box gel doc system (Syngene, United Kingdom).

**TABLE 1:** Primary antibodies in the study.

**Antibody**	**Brand Name**	**Product code**	**Source**	**Dilutability**
anti-CD146 antibody	Abcam	ab75769	Cambridge, United Kingdom	1:1000
anti-SPD antibody	Abcam	ab220422	Cambridge, United Kingdom	1:1000
anti-GAPDH antibody	Cell Signaling Technology	#5174	Beverly, MA, United States	1:1000
anti-AHR antibody	Proteintech	17840-1-AP	Chicago, IL, United States	1:1000
anti-ARNT antibody	Proteintech	14105-1-AP	Chicago, IL, United States	1:1000
anti-ARNT2 antibody	Proteintech	12810-1-AP	Chicago, IL, United States	1:1000

### Immunofluorescence

MLE-12 cells were seeded in glass-bottom dishes (Thermo, United States) and cultured in a 5% CO_2_ atmosphere at 37°C until the cells grew into a single layer. After treatment with PM_2_._5_ or PBS for 12 h, MLE-12 cells were washed three times in PBS and then fixed in 4% paraformaldehyde at 4°C for 15 min. After washing three times with PBS, the cells were blocked with 5% goat serum for 1 h at room temperature. Then, the MLE-12 cells were incubated with a rabbit anti-CD146, rabbit anti-SPD, or rabbit anti-AHR primary antibody at 4°C overnight. After washing three times with PBS, the cells were incubated with Alexa Fluor 555 donkey anti-mouse IgG (H + L) or Alexa Fluor 647 donkey anti-rabbit IgG (H + L) at 37°C for 1 h in the dark. Then, the cells were washed three times with PBS and were stained with DAPI (4′,6-diamidino-2-phenylindole; Yeasen, China) at 37°C for 10 min in the dark. Cellular location and quantification of SPD, CD146, or AHR were visualized using a ZEISS LSM710 confocal fluorescence microscope (Zeiss, Jena, Germany) or an Olympus IX73 fluorescence microscope (Olympus, Tokyo, Japan).

### PAS Staining

After bronchoalveolar lavage in mice, the chest was opened, and the right atrium was flushed with PBS until the lungs turned white. Lung tissues were fixed in 4% paraformaldehyde and embedded in paraffin. Tissue sections were immersed in 0.1% periodic acid for 10 min and washed with distilled water for 5 min. Then the sections were immersed in Schiff’s reagent for 15 min. After washing for 5 min, the sections were again immersed in Mayer’s hematoxylin for 2 min. Finally, tissue sections were dehydrated with ethanol at different concentrations and became transparent with xylene (Dadaci et al., 2015; Hazra et al., 2019). Images of pulmonary sections were visualized using a Zeiss Axio Examiner microscope (Zeiss, Jena, Germany).

### ELISA

Bronchoalveolar lavage fluid of mice was collected and centrifuged to collect the supernatant. The lungs of mice were ground and centrifuged to collect the supernatant. TNF-α, IL-10, and IL-1β are closely related to infection with *C. neoformans* (Maffei et al., 2004). The concentrations of TNF-α (88-7324-22, Invitrogen, United States), IL-10 (431405, BioLegend, United States), and IL-1β (88-7013-22, eBioscience, United States) in BALF and lung homogenates were measured using ELISA kits. Then the concentrations of IL-4 (431104, BioLegend, United States), IL-5 (431204, BioLegend, United States) and IL-13 (900-K207, PeproTech, United States) in lung homogenates were measured using ELISA kits.

### Statistical Analysis

The data are displayed as the mean ± standard error of the mean (SEM). The P-P plot from SPSS 19.0 (IBM Corp., Armonk, NY, United States) was used to test the normal distribution. Data between two groups were analyzed by Student’s *t*-test, or among more than two groups, data were analyzed by one-way ANOVA with Turkey correction using GraphPad Prism 7 software (San Diego, CA). *P* < 0.05 was considered as statistically significant (^∗^*P* < 0.05; ^∗∗^*P* < 0.01; ^∗∗^*P* < 0.001; ns: not significant).

## Results

### PM_2_._5_ Promoted Adhesion of *Cryptococcus neoformans* to Epithelial Cells

**FIGURE 1:**
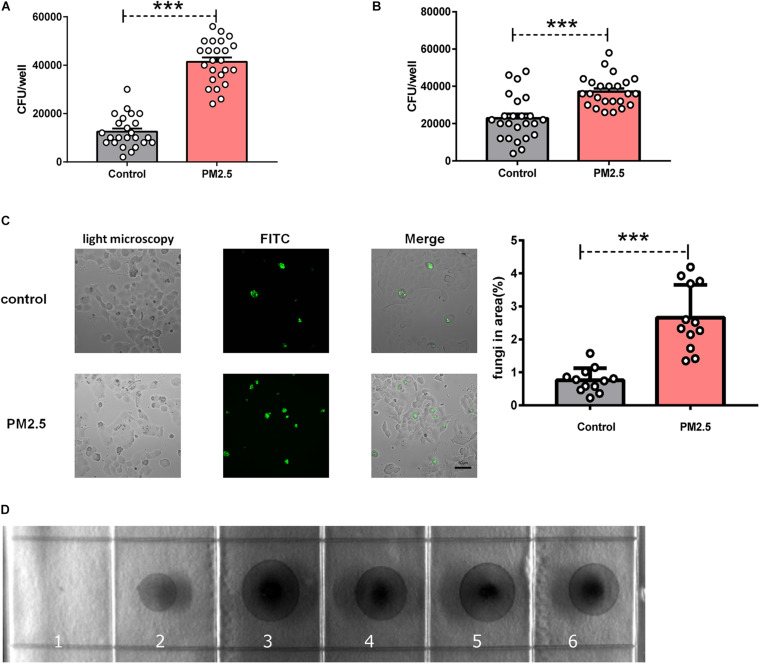
Particulate matter (PM_2_._5__)_ promoted the adhesion of *Cryptococcus neoformans* to epithelial cells. **(A)** CFU analysis of *Cryptococcus neoformans* adhered to mouse pulmonary epithelial cell line (MLE-12) cells, control: MLE-12 cells pretreated with PBS for 12 h, *n* = 24; PM_2_._5_ MLE-12 cells pretreated with 10 μg/ml PM_2_._5_ for 12 h, *n* = 24. **(B)** CFU analysis of *Cryptococcus neoformans* adhered to primary pulmonary epithelial cells, control: primary pulmonary epithelial cells treated with PBS for 12 h, *n* = 24; PM_2_._5_: primary pulmonary epithelial cells treated with 10 μg/ml PM_2_._5_ for 12 h, *n* = 24. **(C)** Immunofluorescence analysis of *Cryptococcus neoformans* adhered to MLE-12 cells, control: MLE-12 cells treated with PBS for 12 h, *n* = 12; PM_2_._5_: MLE-12 cells treated with 10 μg/ml PM_2_._5_ for 12 h, *n* = 12; the analysis of fungi in area (%) is presented. **(D)** CFU analysis of *Cryptococcus neoformans* in the collected PM_2_._5_, 1: No PM_2_._5_ was plated onto Sabouraud dextrose agar plates; 2: PM_2_._5_ (10 μl, 10 μg/ml) was plated onto Sabouraud dextrose agar plates; 3–6: PM_2_._5_ (20 μl, 10 μg/ml) was plated onto Sabouraud dextrose agar plates. Student’s *t*-test was performed after the normal distribution analysis. ***P < 0.001.

To explore whether PM_2_._5_ increased fungal infection, we treated epithelial cells with PM_2_._5_ prior to *C. neoformans* infection. After extensively washing, free and loose fungal cells were washed away, still *C. neoformans* recovered from PM_2_._5_-treated MLE-12 cells formed more CFUs (Figure 1A), which was also validated in primary alveolar epithelial cells (Figure 1B). The epithelial cell line is the first barrier of defense and may kill pathogens through diverse molecules (Schleimer et al., 2007). To directly demonstrate whether PM_2_._5_ increased the adhesion of *C. neoformans*, fungal cells attached to the epithelial monolayer were directly observed and recorded under a light microscope. Indeed, *C. neoformans* were more likely to attach to PM_2_._5_-treated MLE-12 cells than to untreated cells (Figure 1C). To explore whether PM_2_._5_ was contaminated with fungi, we inoculated PM_2_._5_ on Sabouraud dextrose agar plates and cultured at 32°C for 48 h. We could not identify any fungi colony in the plate (Figure 1D), suggesting that PM_2_._5_ itself did not contain *C. neoformans* and other fungi. Of note, *C. neoformans* may be internalized by epithelial cells, which may be mistaken for the adhesion. However, as reported in the brain endothelial cells, adhered fungi were the major form of *C. neoformans* attached to the barrier cells (Sabiiti and May, 2012). In summary, PM_2_._5_ promoted the adhesion of *C. neoformans* to alveolar epithelial cells.

### *Cryptococcus neoformans* Capsule and Viability Affected Fungal Adhesion

**FIGURE 2:**
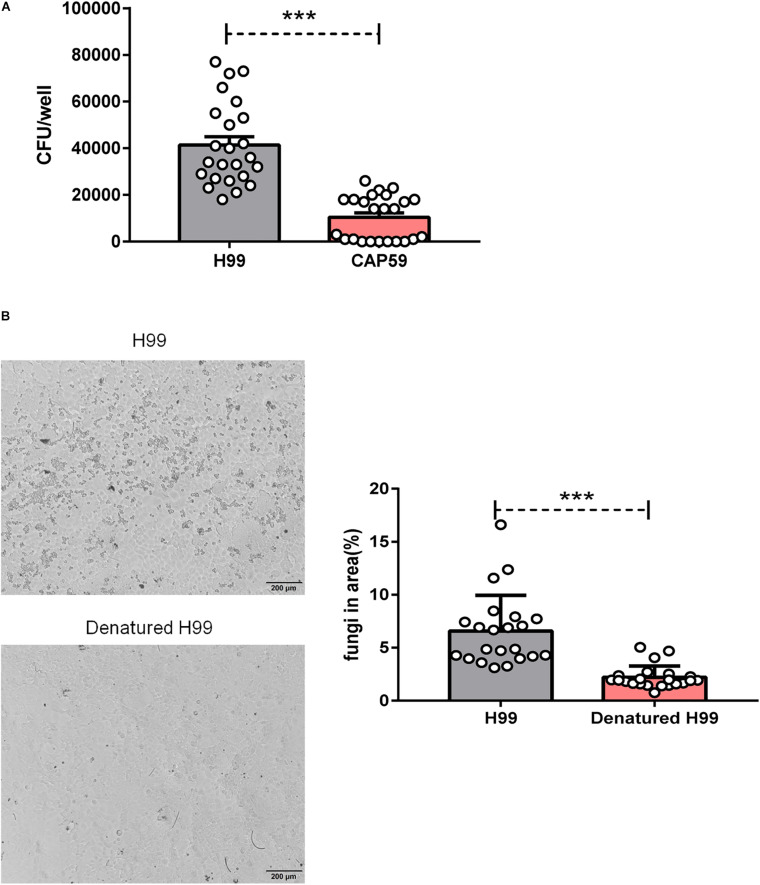
*Cryptococcus neoformans* capsule and viability affected fungal adhesion. **(A)** CFU analysis of wild-type strain H99 and acapsular strain CAP59 adhered to MLE-12 cells treated with 10 μg/ml PM_2_._5_ for 12 h. **(B)** Light microscopy analysis of undenatured H99 and denatured H99 adhered to MLE-12 cells treated with 10 μg/ml PM_2_._5_ for 12 h (H99 is smaller and rounder relative to the MLE-12 cells) and the analysis of fungi in the area (%) is presented. Student’s *t*-test was performed after the normal distribution analysis. ***P < 0.001.

The capsule is the major virulence component of *C. neoformans* (O’Meara and Alspaugh, 2012). To dissect the roles of capsule in PM_2_._5_-mediated adhesion, we infected epithelial cells with *C. neoformans* encapsulated strain H99 or the capsular mutant strain CAP59. As shown in Figure 2A, the *C. neoformans* encapsulated strain H99 developed more CFU than acapsular strain CAP59 on the PM_2_._5_-treated MLE-12 cells, implying that the capsule may be indispensable for PM_2_._5_-boosted *C. neoformans* infection.

The reduced *C. neoformans* infection from the acapsular strain may be due to the decreased viability of the capsule-deficient strain or a direct reduction in adhesion. To explore the roles of fungal viability in the PM_2_._5_-boosted *C. neoformans* infection, we challenged PM_2_._5_-treated epithelial cells with heat-killed *C. neoformans*. As expected, the adhesion of killed fungi on epithelial cells almost disappeared under light microscopy observation (Figure 2B). Collectively, these results showed that *C. neoformans* adhesion on PM_2_._5_-treated epithelial cells may be regulated by fungal capsule and viability.

### PM_2_._5_ Increased Surfactant Protein D and CD146 Expression on Alveolar Epithelial Cells

**FIGURE 3:**
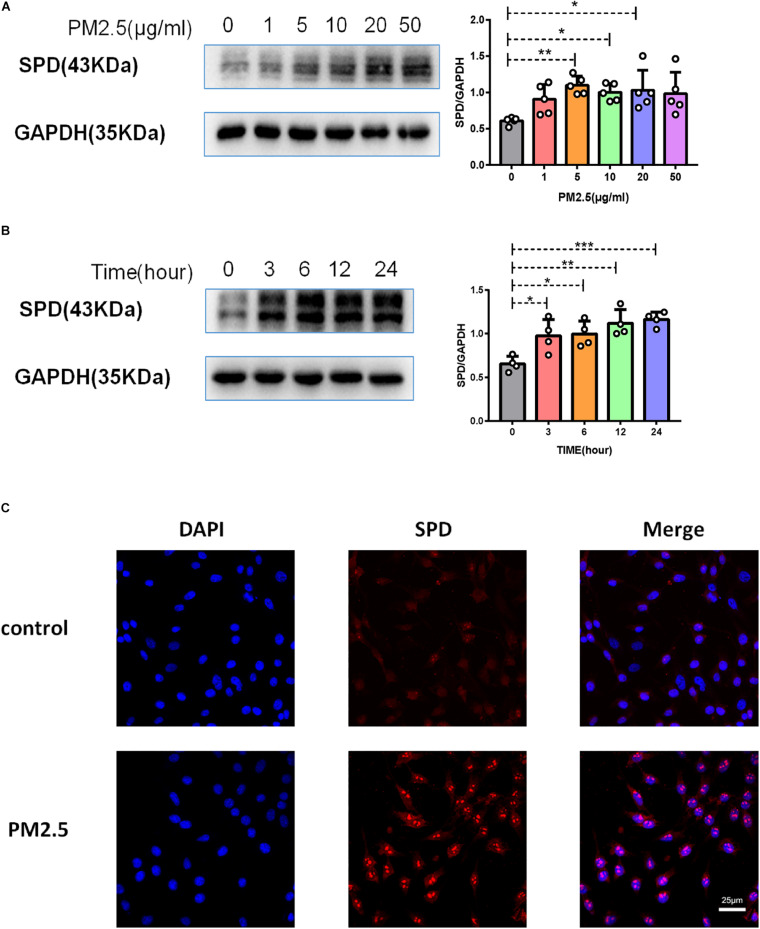
PM_2_._5_ increased surfactant protein D (SPD) expression on alveolar epithelial cells. **(A)** Western blot analysis of SPD in MLE-12 cells treated with PM_2_._5_ (0, 1, 5, 10, 20, or 50 μg/ml) for 24 h. **(B)** Western blot analysis of SPD in MLE-12 cells treated with PM_2_._5_ (10 μg/ml) for 0, 3, 6, 12, or 24 h. **(C)** Immunofluorescence analysis of CD146 in MLE-12 cells, control: MLE-12 cells treated with PBS for 12 h; PM_2_._5_: MLE-12 cells treated with 10 μg/ml PM_2_._5_ for 12 h. *P < 0.05; **P < 0.01; ***P < 0.001.

**FIGURE 4:**
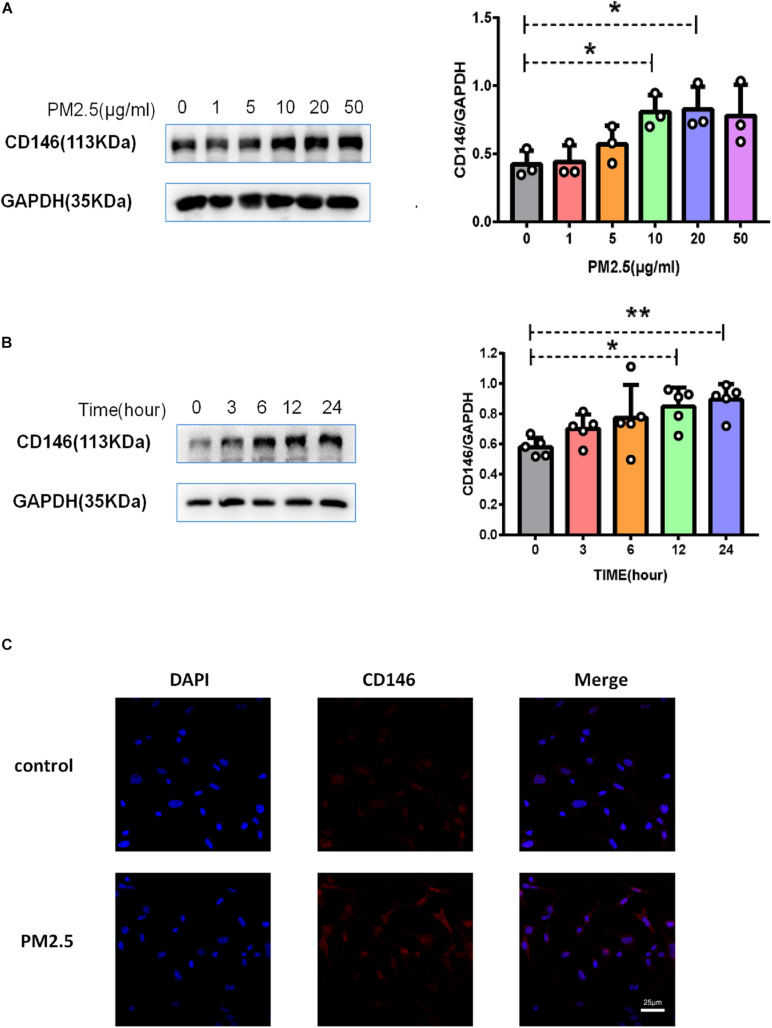
PM_2_._5_ increased CD146 expression on alveolar epithelial cells. **(A)** Western blot analysis of CD146 in MLE-12 cells treated with PM_2_._5_ (0, 1, 5, 10, 20, or 50 μg/ml) for 24 h. **(B)** Western blot analysis of CD146 in MLE-12 cells treated with PM_2_._5_ (10 μg/ml) for 0, 3, 6, 12, or 24 h. **(C)** Immunofluorescence analysis of CD146 in MLE-12 cells, control: MLE-12 cells treated with PBS for 12 h; PM_2_._5_: MLE-12 cells treated with 10 μg/ml PM_2_._5_ for 12 h. *P < 0.05; **P < 0.01.

As surfactant protein D (SPD) promoted the adhesion of *C. neoformans* on epithelial cells (Geunes-Boyer et al., 2012), we first evaluated the roles of PM_2_._5_ on the expression of SPD in epithelial cells. As expected, the protein expression of SPD was increased in MLE-12 cells treated with PM_2_._5_ (Figures 3A,B), which was also directly observed under a fluorescence microscope (Figure 3C). Similar to the change in SPD expression, CD146 expression was increased in epithelial cells treated with PM_2_._5_ in a dose-dependent manner; 10 μg/ml PM_2_._5_ was most effective to significantly increase CD146 expression in 12 h (Figures 4A,B). Immunofluorescence analysis indicated that CD146 expression in MLE-12 cells was not only restricted to cell membrane but also in the cytoplasm, which was upregulated with PM_2_._5_ (Figure 4C). In summary, PM_2_._5_ increased the expression of the adhesion molecules SPD and CD146 on alveolar epithelial cells.

### CD146 Expression Was Dependent on Aryl Hydrocarbon Receptor/ARNT Signaling

**FIGURE 5:**
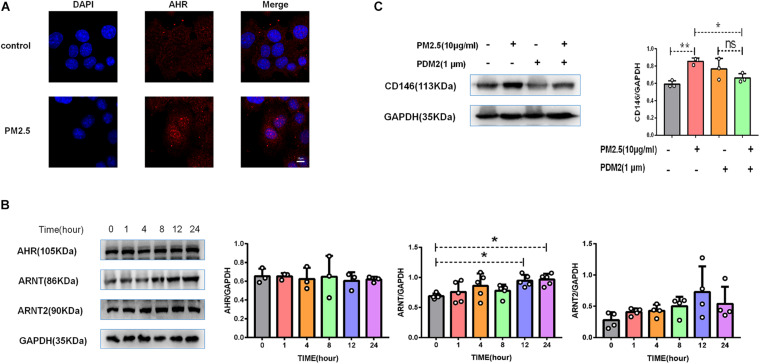
CD146 expression was dependent on aryl hydrocarbon receptor (AhR)/ARNT signaling. **(A)** Immunofluorescence analysis revealed that AhR was translocated into the nucleus in MLE-12 cells treated with PM_2_._5_, control: MLE-12 cells treated with PBS for 12 h; PM_2_._5_: MLE-12 cells treated with 10 μg/ml PM_2_._5_ for 12 h. **(B)** Western blot analysis of AhR, ARNT, and ARNT2 in MLE-12 cells treated with 10 μg/ml PM_2_._5_. **(C)** Western blot analysis of CD146 in MLE-12 cells pretreated with 1 μm AHR inhibitor (PDM2, MedChemExpress, HY-112629) for 1 h and challenged with 10 μg/ml PM_2_._5_ for 12 h. *P < 0.05; **P < 0.01; ns, not significant.

Previously, we reported that PM_2_._5_ collected in city streets contained various PAHs. Based on the HPLC results, the total PAHs in PM_2_._5_ was 156.78 μg/g, including chrysene, benzo[a]anthracene, and others (Chen et al., 2018). PM_2_._5_ promoted lung cancer metastasis via aryl hydrocarbon receptor (AhR) signaling (Chen et al., 2018). Similarly, upon PM_2_._5_ treatment, AhR was translocated into the nucleus (Figure 5A), implying activation of the AhR pathway (Davarinos and Pollenz, 1999). In the nucleus, AhR dimerized with its chaperone ARNT or ARNT2 to mediate gene regulation. In MLE-12 cells, PM_2_._5_ increased the expression of ARNT but not ARNT2 (Figure 5B), implying that ARNT may be dominant in AhR signaling in epithelial cell response to PM_2_._5_. Moreover, an AhR inhibitor antagonized the upregulation of CD146 induced by PM_2_._5_ (Figure 5C). Therefore, CD146 expression in alveolar epithelial cells treated with PM_2_._5_ was at least partially dependent on AhR/ARNT signaling.

### CD146 Mediated *Cryptococcus neoformans* Adhesion to Epithelial Cells

**FIGURE 6:**
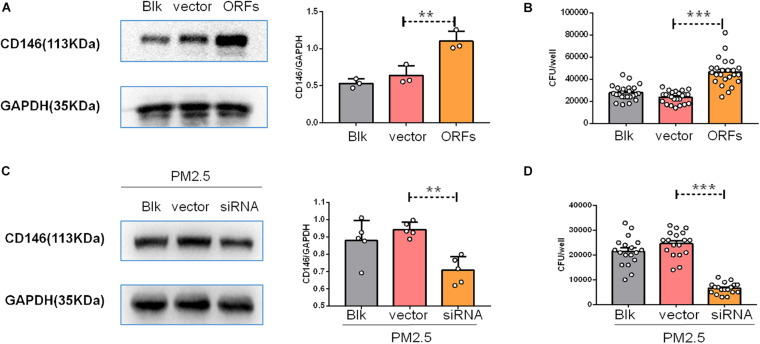
CD146 mediated *Cryptococcus neoformans* adhesion to epithelial cells. **(A)** Western blot analysis of CD146 in MLE-12 cells added with blank plasmid (Blk) or a CD146 expression plasmid (CD146 open reading frame, CD146 ORF). **(B)** CFU analysis of *Cryptococcus neoformans* adhered to MLE-12 added with blank plasmid or CD146 ORF plasmid. **(C)** Western blot analysis of CD146 in MLE-12 cells added with blank plasmid or a CD146 siRNA plasmid before MLE-12 treated with 10 μg/ml PM_2_._5_ for 12 h. **(D)** CFU analysis of *Cryptococcus neoformans* adhered to MLE-12 cells added with blank plasmid or a CD146 siRNA plasmid before MLE-12 treated with 10 μg/ml PM_2_._5_ for 12 h. Student’s *t*-test was performed after the normal distribution analysis. **P < 0.01; ***P < 0.001.

CD146 contributed to bacterial adherence to the respiratory tract (Simon et al., 2011), suggesting that CD146 may be an adhesion molecule for pathogens. To directly explore the roles of CD146 in fungal cell adhesion, we overexpressed CD146 with expression plasmid (Figure 6A); *C. neoformans* adhesion on MLE-12 cells accordingly increased (Figure 6B). Previously, we demonstrated that PM_2_._5_ increased CD146 expression on alveolar epithelial cells. Herein, we silenced CD146 expression with CD146 siRNA plasmid (Figure 6C). In the CD146-silenced epithelial cells, *C. neoformans* adhesion on PM_2_._5_-treated MLE-12 cells was significantly decreased (Figure 6D). Collectively, these results suggested that CD146 mediated the adhesion of *C. neoformans* to epithelial cells.

### PM_2_._5_ Promoted *Cryptococcus neoformans* Infection *in vivo*

**FIGURE 7:**
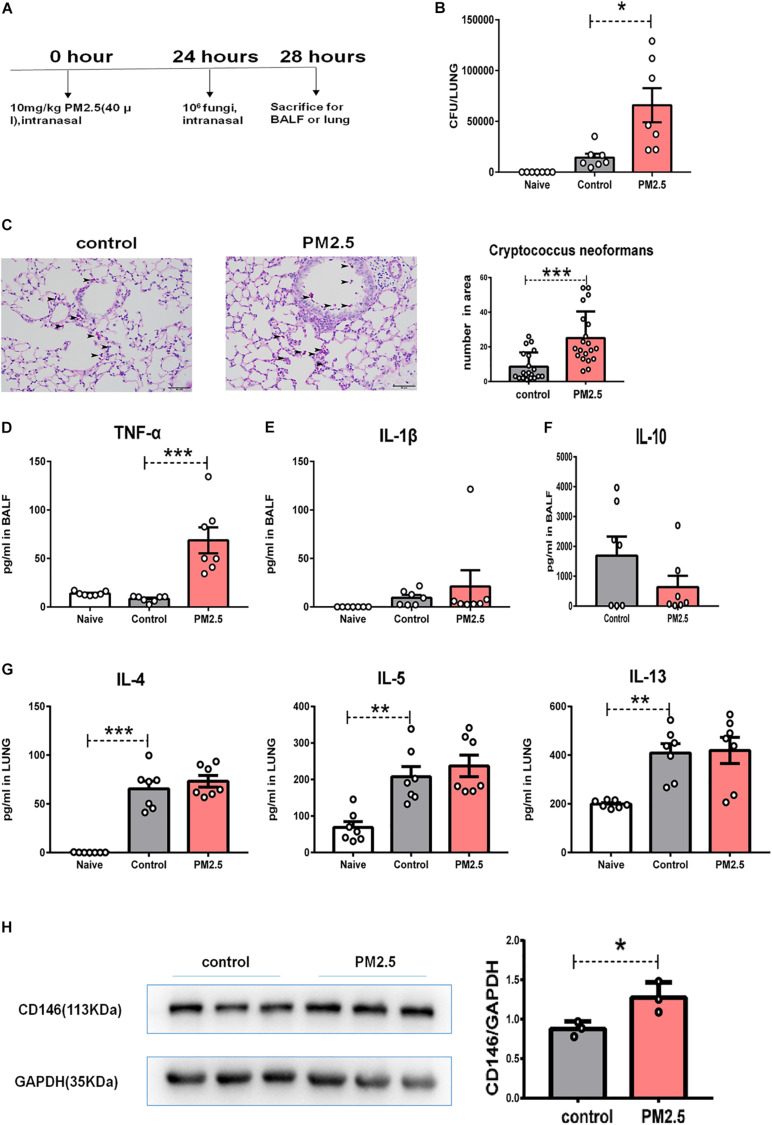
PM_2_._5_ promoted *Cryptococcus neoformans* pulmonary infection. **(A)** Flow chart of the *Cryptococcus neoformans* infection model. **(B)** CFU analysis of *Cryptococcus neoformans* in the lungs. Naïve: mice treated without PM_25_ and *Cryptococcus neoformans*, *n* = 7. Control: mice treated with PBS and *Cryptococcus neoformans*, *n* = 7; PM_2_._5_: mice treated with 10 mg/kg PM_2_._5_ and *Cryptococcus neoformans*; *n* = 7. **(C)** Representative images of lung sections stained with PAS, arrow indicated the fungi. **(D)** ELISA analysis of TNF-α in bronchoalveolar lavage fluid (BALF); *n* = 7. **(E)** ELISA analysis of IL-1β in BALF; *n* = 7. **(F)** ELISA analysis of IL-10 in BALF; *n* = 7. **(G)** ELISA analysis of IL-4, IL-5, and IL-13 in the lungs; *n* = 7. **(H)** Western blot analysis of CD146 in the lungs. Student’s *t*-test was performed after the normal distribution analysis. *P < 0.05; **P < 0.01; ***P < 0.001.

To provide direct evidence for the roles of PM_2_._5_ in fungal infection, we infected PM_2_._5_-treated mice with *C. neoformans* (Figure 7A). After 4 h of exposure to fungal cells, the mice were sacrificed, and free fungal cells were cleared from the respiratory tracts with thorough perfusion. As shown in Figure 7B, naïve mice without the inoculation with *C. neoformans* were free from fungi in the lung; compared with the control mice with *C. neoformans* infection, pulmonary fungal burden was significantly increased in the PM_2_._5_-treated and *C. neoformans*-infected mice. According to PAS staining, more fungal cells were recorded in the alveoli from PM_2_._5_-treated mice than in those from untreated mice (Figure 7C), suggesting that PM_2_._5_ may increase fungal adhesion and pulmonary infection.

As PM_2_._5_ may cause inflammatory response (Zhu et al., 2019; Sun et al., 2020), we measured TNF-α and IL-1β in BALF. As shown in Figures 7D,E, PM_2_._5_ augmented levels of the inflammatory cytokine TNF-α in the *C. neoformans* lung infection. *C. neoformans* not only induced the production of TNF-α and IL-1β but also promoted the anti-inflammatory cytokine IL-10 (Maffei et al., 2004). However, IL-10 was comparable in the *C. neoformans* infected mice pretreated with or without PM_2_._5_ (Figure 7F). Type 2 cytokines (IL-4, IL-5, and IL-13) were essential for the *C. neoformans* chronic infection (Dutra et al., 2018). As expected, *C. neoformans* inoculation increased IL-4, IL-5, or IL-13 in pulmonary homogenates (Figure 7G); however, PM_2_._5_ was insignificant in the type 2 cytokine production (Figure 7G). CD146 expression, however, was upregulated in the *C. neoformans-*infected mice pretreated with PM_2_._5_ (Figure 7H), raising the possibility that CD146 may mediate *C. neoformans* pulmonary infection *in vivo*. In summary, PM_2_._5_ promoted *C. neoformans* infection in the lungs and increased the expression of CD146.

### CD146 Deficiency Decreased *Cryptococcus neoformans* Infection in the PM_2_._5_-Treated Mice

**FIGURE 8:**
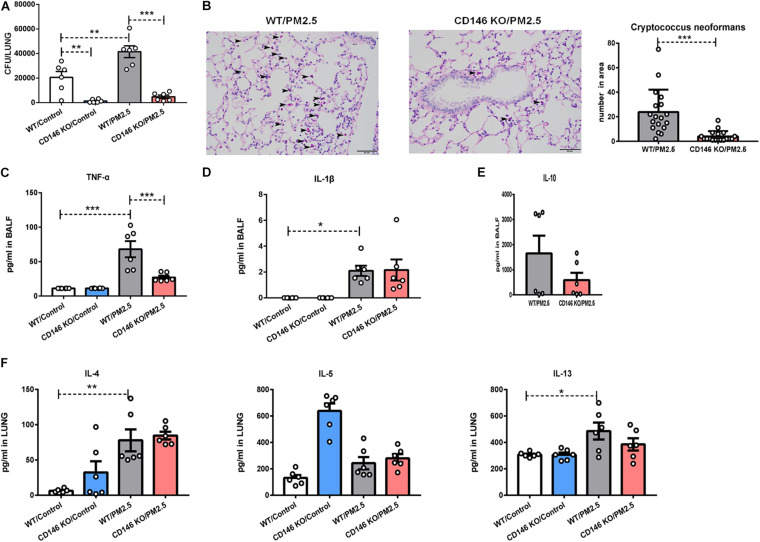
CD146 deficiency decreased *Cryptococcus neoformans* infection. **(A)** CFU analysis of *Cryptococcus neoformans* in the lungs. WT, wild type, *n* = 6; CD146 KO: CD146 Knock out, *n* = 6; PM_2_._5_ group: mice were pretreated with PM_2_._5_ (10 mg/kg) 24 h before *Cryptococcus neoformans* infection. Control group: mice were pretreated with PBS 24 h before *Cryptococcus neoformans* infection. All mice were sacrificed 4 h post fungal infection. **(B)** Representative images of lung sections stained with PAS; arrow indicates the fungi. **(C)** ELISA analysis of TNF-α in BALF; *n* = 6. **(D)** ELISA analysis of IL-1β in BALF; *n* = 6. **(E)** ELISA analysis of IL-10 in BALF; *n* = 6. **(F)** ELISA analysis of IL-4, IL-5, and IL-13 in the lungs; *n* = 6. Student’s *t*-test was performed after the normal distribution analysis. *P < 0.05; **P < 0.01; ***P < 0.001.

To further demonstrate the roles of CD146 in the infection of *C. neoformans*, we pretreated wild-type (WT) mice and CD146 knockout (KO) mice with PM_2_._5_ for 24 h and infected these mice with fungi. As shown in Figure 8A, the fungal burden in CD146-deficient mice was significantly diminished in the control or PM_2_._5_ group. Accordingly, PAS-positive stained fungal cells were also decreased in the alveoli from CD146-deficient mice (Figure 8B). There was no significant difference in the expression of TNF-α and IL-1β between the WT or CD146 KO mice without PM_2_._5_ stimulation. In contrast, TNF-α was reduced in CD146 KO mice treated with PM_2_._5_ (Figure 8C). Meanwhile, anti-inflammatory cytokine IL-10 and type 2 cytokines (IL-4, IL-5, and IL-13) were similar in the WT or CD146 KO mice with fungal infection (Figures 8D–F). Collectively, CD146 deficiency decreased inflammatory response and *C. neoformans* infection in the PM_2_._5_-treated mice.

## Discussion

Adhesion is the first step in establishing an infection. Host cells express various functional receptors, which are deliberately utilized by pathogens for attachment. In the present study, we provided evidences that the air pollutant PM_2_._5_ increased the expression of adhesion molecule CD146 on alveolar epithelial cells in an AhR-dependent pathway. *In vitro*, CD146 expression was in line with the adhesion of *C. neoformans* to alveolar epithelial cells. In a murine model of *C. neoformans* pulmonary infection, PM_2_._5_ promoted fungal infection, and CD146 deficiency significantly impaired the adhesion of fungal cells to the respiratory tract.

As an adhesion molecule, CD146 on epithelial cells mediates the adherence of bacteria to the airway epithelium (Simon et al., 2011). In virus infection, CD146 on epithelial cells is indispensable for inflammatory cytokine IL-8 production, thereby amplifying inflammation (Berman et al., 2014). IL-8 homologs in rodents included CXCL1/KC, CXCL2/MIP-2, and CXCL5-6/LIX (Hol et al., 2010). In the murine model of *C. neoformans* pulmonary infection, CD146 deficiency was accompanied by a reduction in the levels of the pro-inflammatory cytokines TNF-α and IL-1β. In addition to epithelial cells, macrophages and other immune cells also express CD146, which is involved in the regulation of the immune response against bacterial (Wu et al., 2013) and viral infections (Berman et al., 2014, 2016). In a cell culture experiment, we clearly showed that CD146 expression was involved in fungal adhesion. However, we could not preclude the possibility that the fungal burden reduction in the CD146 silenced epithelial cells or in the CD146-deficient mice may be beyond the adhesion function of CD146. *C. neoformans* manipulated CD14 (Barbosa et al., 2007) and SPD (Geunes-Boyer et al., 2012) for cell adhesion receptor in respiratory epithelial cells. We speculated that these receptors may work in coordination with *C. neoformans* adhesion and infection.

In the present study, capsule deficiency or heat killing decreased the adhesion of fungal cells to epithelial cells. Roles of capsule in *C. neoformans* adhesion to host cells were arguable. *C. neoformans* capsule major component glucuronoxylomannan (GXM) directly bound with CD14 in epithelial cells (Barbosa et al., 2007) directly mediated adhesion (Barbosa et al., 2006), in line with our observation that capsule deficiency decreased fungal cell adhesion. However, other studies indicated that acapsular *C. neoformans* adhered to epithelial cells more effectively (Merkel and Scofield, 1997; Choo et al., 2015). It is postulated that wild-type *C. neoformans* exploit GXM for adherence to epithelial cells, and the acapsular mutants depended on mannoprotein 84 (MP84) for adhesion (Teixeira et al., 2014). Free capsule components and the capsule recovered strain should be included in future analyses in hoping to explore the interactions between fungal components and the adhesion receptors.

Exposure to PM_2_._5_ has been associated with increased lung infections (Kim et al., 2015). Even short-term PM_2_._5_ exposure was positively associated with acute lung infection (Nhung et al., 2018; Kim et al., 2020) and COPD exacerbation (Tian et al., 2018). Mechanically, PM_2_._5_ may directly bring inhalable bacteria into the respiratory system (Cao et al., 2014). PM_2_._5_ in the present study was negative for fungal cell contamination. However, PM_2_._5_ from *C. neoformans*-contaminated soil is capable of transporting fungi into the lungs. In addition, PM_2_._5_ may compromise immune cells, thereby reducing immune defense against pathogens (Zhao et al., 2014; Harkema et al., 2017). Our study adds the possibility that PM_2_._5_ may promote the adhesion of pathogens to the respiratory epithelium via the upregulated receptor CD146, which may shed light on the acquisition of pulmonary fungal infections.

In summary, our study provided the proof that air pollution PM_2_._5_ may promote pneumonia caused by environmental fungus *C. neoformans* via the upregulation of adhesion molecule CD146. This study raised the possibility of increased risk of elusive fungi pneumonia upon exposure to polluted air, and CD146 may be a potential target in the prevention of pulmonary cryptococcosis.

## Data Availability Statement

All datasets generated for this study are included in the article/supplementary material.

## Ethics Statement

The animal study was reviewed and approved by the Nanjing Medical University Ethics Committee.

## Author Contributions

NJ, MH, and MZ designed the experiments. ZS, JJ, ZW, and ZC performed the animal experiments. YT, EZ, and XY performed the cell experiments. NJ and ZS drafted the manuscript. MZ edited and revised the manuscript. All authors contributed to the article and approved the submitted version.

## Conflict of Interest

The authors declare that the research was conducted in the absence of any commercial or financial relationships that could be construed as a potential conflict of interest.
